# CrMPK3, a mitogen activated protein kinase from *Catharanthus roseus* and its possible role in stress induced biosynthesis of monoterpenoid indole alkaloids

**DOI:** 10.1186/1471-2229-12-134

**Published:** 2012-08-07

**Authors:** Susheel Kumar Raina, Dhammaprakash Pandhari Wankhede, Monika Jaggi, Pallavi Singh, Siddhi Kashinath Jalmi, Badmi Raghuram, Arsheed Hussain Sheikh, Alok Krishna Sinha

**Affiliations:** 1 National Institute of Plant Genome Research (NIPGR), Aruna Asaf Ali Road, New Delhi, 110067, India

**Keywords:** *Catharanthus roseus*, Methyl jasmonate, Mitogen activated protein kinase, Monoterpenoid indole alkaloid, Secondary metabolism

## Abstract

**Background:**

Mitogen activated protein kinase (MAPK) cascade is an important signaling cascade that operates in stress signal transduction in plants. The biologically active monoterpenoid indole alkaloids (MIA) produced in *Catharanthus roseus* are known to be induced under several abiotic stress conditions such as wounding, UV-B etc. However involvement of any signaling component in the accumulation of MIAs remains poorly investigated so far. Here we report isolation of a novel abiotic stress inducible *Catharanthus roseus* MAPK, *CrMPK3* that may have role in accumulation of MIAs in response to abiotic stress.

**Results:**

CrMPK3 expressed in bacterial system is an active kinase as it showed auto-phosphorylation and phosphorylation of Myelin Basic Protein. CrMPK3 though localized in cytoplasm, moves to nucleus upon wounding. Wounding, UV treatment and MeJA application on *C. roseus* leaves resulted in the transcript accumulation of *CrMPK3* as well as activation of MAPK in *C. roseus* leaves. Immuno-precipitation followed by immunoblot analysis revealed that wounding, UV treatment and methyl jasmonate (MeJA) activate CrMPK3*.* Transient over-expression of *CrMPK3* in *C. roseus* leaf tissue showed enhanced expression of key MIA biosynthesis pathway genes and also accumulation of specific MIAs.

**Conclusion:**

Results from our study suggest a possible involvement of *CrMPK3* in abiotic stress signal transduction towards regulation of transcripts of key MIA biosynthetic pathway genes, regulators and accumulation of major MIAs.

## Background

Plants in order to cope-up with changing environmental conditions synthesize a wide variety of secondary metabolites, which not only acts as defence molecules but also contribute to its overall growth and development. Biosynthesis of these compounds is often induced by various environmental stimuli and stress factors such as UV light or pathogen attack. *Catharanthus roseus* (L.) G. Don, a tropical plant species, synthesizes more than 130 monoterpenoid indole alkaloids (MIAs) as part of its secondary metabolism. Some of the MIAs possess high therapeutic value such as antineoplastic drugs vinblastine and vincristine, so have earned a great commercial importance [1]. These MIAs are produced at very low levels via a complex MIA biosynthetic pathway that is also reported to be stress induced in *C. roseus*. However, factors such as fungal elicitors, heavy metal ions, UV radiation, osmotic shock, wounding or pathogen attack induce their biosynthesis. Treatment of *C. roseus* seedlings with methyl jasmonate (MeJA) increases the activity levels of TDC (tryptophan decarboxylase), STR (Strictosidine synthase), D4H (Desacetoxyvindoline −4-hydroxylase) and DAT (Deacetylvindoline 4-O-acetyltransferasee) and leads to enhanced accumulation of vindoline [2]. Moreover, elicitor induced JA biosynthesis and MeJA induced *Tdc* and *Str* are blocked by K-252a, a protein kinase inhibitor suggesting involvement of protein phosphorylation in this signal transduction [3]. To date there is no information regarding the involvement of any signaling component towards the accumulation of alkaloids in response to stress. One can speculate the involvement of mitogen activated protein kinase (MAPK) cascade since wounding and systemin have been known to activate MAPKs upstream of octadecanoid pathway in tomato plants as well as autotrophic cell cultures [4]. In Arabidopsis, regulation of camalexin biosynthesis by MPK3/MPK6 cascade has been reported [5] MAP kinase is one of the major signaling cascades by which extracellular stimuli are transduced into intracellular responses [6]. MAPK cascade includes three functionally linked kinases: MAP Kinase (MAPK); MAPK kinase (MAPKK) and MAPKK kinase (MAPKKK). Upon elicitation by external stimuli, the receptors mediate the phosphorylation and activation of MAPKKK. This activated MAPKKK activates MAPKK by phoshorylation at serine and threonine residues, which in turn activates MAPK by phosphorylation at threonine and tyrosine residues [7]. MAPKs are known to be activated by a variety of biotic and abiotic [6-9] stresses in plants. In mammals and yeast, MAPK cascades are active downstream to G-protein coupled receptors, receptor tyrosine kinases (RTKs) or two component histidine kinases. Most of the MAPK substrates include transcription factors, transcription regulators, splicing factors, receptors, histones and others [10]. The role of MAPKs has been implicated in various biological phenomena in plants including pathogen defense, abiotic stresses, cytokinesis and cell differentiation, and plant hormone signaling [7].

In the present study we report cloning of a mitogen activated protein kinase from *C. roseus*, *CrMPK3*. The transcript and activity of CrMPK3 is induced by the same stimuli known to induce alkaloid biosynthesis in *C. roseus.* Further, transient over-expression of *CrMPK3* in *C. roesus* leaves results in up-regulation of specific alkaloids and the transcripts of genes involved in MIA biosynthesis pathway. We infer that CrMPK3 may have its role in stress-mediated accumulation of MIA in *C. roseus*.

## Results

### Methyl jasmonate, UV and wounding activate MAP kinases in *C. roseus*

**Figure 1:**
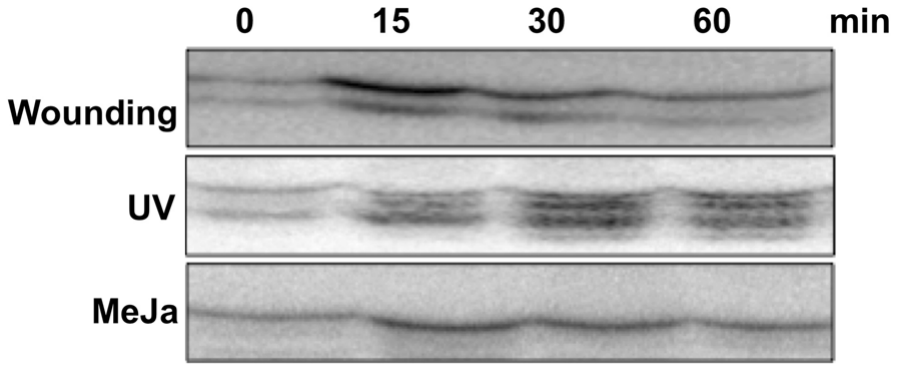
**Activation of MAP Kinases by wounding, UV and MeJA treatment in *C. roseus.*** 6*–*8 weeks old *C. roseus* plants were subjected to various stress treatments. Activation of MAPK in response to wounding, UV treatment, and MeJA treatment was determined by in-gel kinase assay using MBP as a substrate polymerized in SDS 10%(w/v) polyacrylamide gel. Autoradiograms represent in-gel phosphorylation of MBP. The experiments have been repeated three times with similar result.

MAP kinase cascade is known to regulate several stress responsive biological processes in different plants. In order to understand the signaling component of MIA biosynthesis we checked activation of MAPK in conditions that lead to accumulation of MIAs. In *C. roseus* methyl jasmonate treatment [2,11] and stress conditions such as UV [12] and wounding [13] have been shown to enhance alkaloid accumulation. We therefore, used these three conditions to study MAPK activity in *C. roseus* leaves. Equal amounts of protein extracted from the treated leaf tissue were analyzed in an in-gel kinase assay using myelin basic protein (MBP) as an artificial substrate. As shown in Figure 1, a transient activation of two different MAP kinases were observed within 15 minutes of wounding that decreased gradually. UV treatment also leads to increase in kinase activity within 30 minutes of treatment. At least three MBP phosphorylating kinases were observed after UV exposure whereas methyl jasmonate led to activation of single MAPK (Additional file 1: Figure 1). 

### Cloning of *CrMPK3*

Having seen the activation of MAPK in different stress conditions we attempted cloning MAP kinase gene from *C. roseus* that might be regulating MIA pathway. For the purpose, a cDNA library of *C. roseus* in λZAP II was screened by a 438 bp MAPK EST (GenBank accession No. AJ537469). The sequencing of positive plaques obtained after tertiary screening gave a 630 bp fragment that showed maximum identity with *Wound Induced Protein Kinase* (WIPK) from *Nicotiana attenuata* (ABJ89813). However, the identified clone was a partial gene with missing 5′ end. To get full-length MAPK gene, a forward degenerate oligonucleotide was designed from 5' end of several MAPK sequences from other plant species that showed significantly high identity with the 630 bp fragment obtained from library screening. The reverse primer was designed from the 3′ end of the identified partial MAPK clone. PCR amplification of *C. roseus* cDNA with 5′ degenerate and 3′ gene specific primers resulted in an amplicon of about 1.1 Kb. The cloning and sequence analysis of the amplicon led to identification of an ORF of 1119 bp long encoding distinct *C. roseus* MAPK. Since the identified *MAPK* gene shows maximum identity with tobacco WIPK and Arabidopsis AtMPK3 (Additional file 2: Figure 2) we named it as *CrMPK3* (EF156758). Phylogenetic analysis of *CrMPK3* (Additional file 2) indicated that it belongs to group A MAPKs [14] that are involved in signaling pathogen infections and abiotic stresses. Analysis of deduced amino acid sequence revealed that the *CrMPK3* encodes 372 amino acids and the protein contains all the 11 subdomains (Additional file 3) that are conserved among all MAPK families [15]. The ‘TEY’ phosphorylation motif is found between VII and VIII subdomains of CrMPK3. *In silico* analysis of the sequence revealed an approximate molecular weight of 43 kDa. 

**Figure 2:**
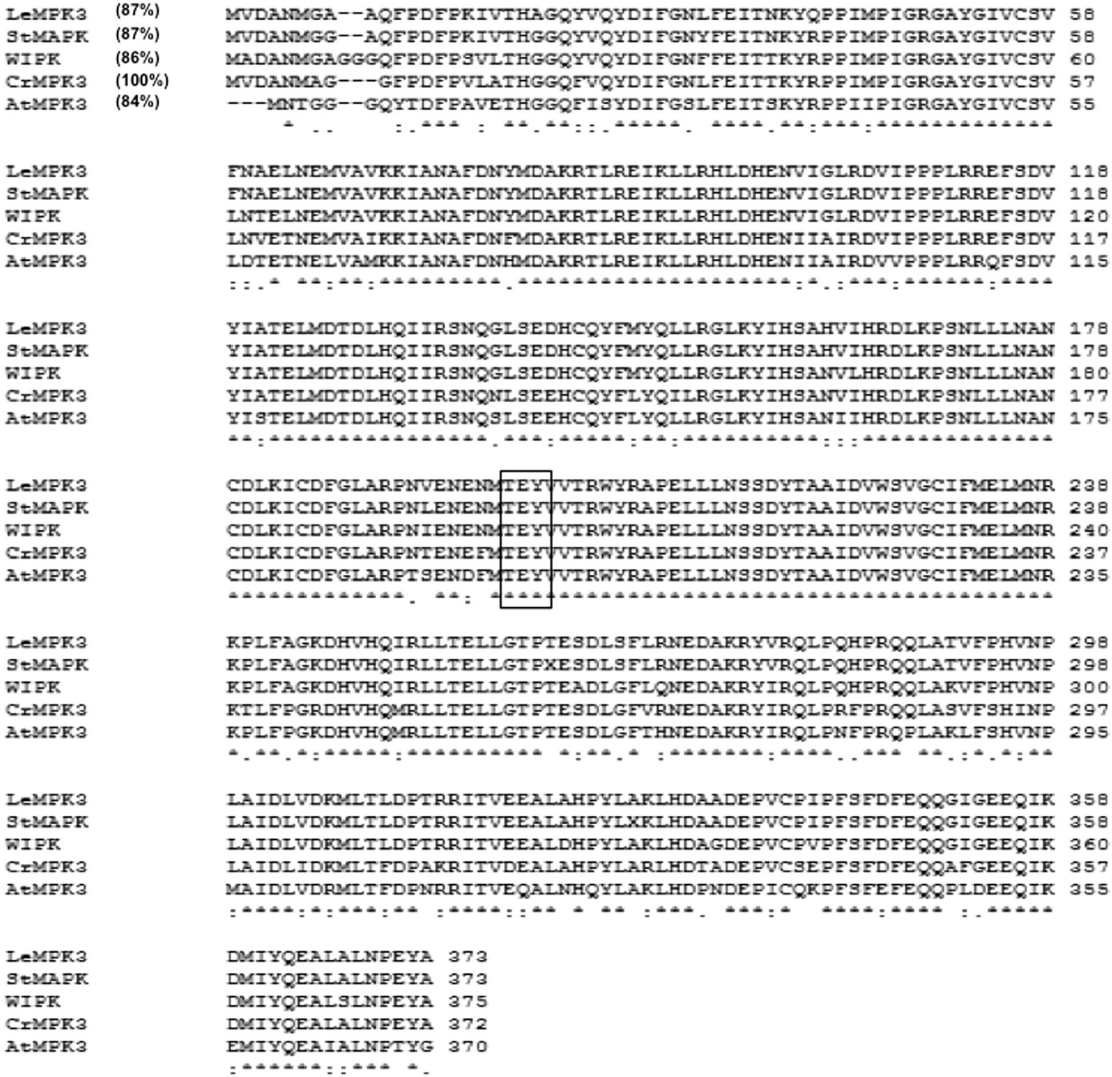
**Multiple sequence alignment of CrMPK3 and related MAPK sequences using ClustalW(1.82). ** The NCBI accession numbers are *Lycopersicon esculentum LeMPK3* (AY261514), *Solanum tuberosum* MAP Kinase, (AB206552), *Nicotiana attenuata WIPK* (DQ991136) and *A. thaliana* AtMPK3 (NM114433).

### Recombinant CrMPK3 is an active MAPK

**Figure 3:**
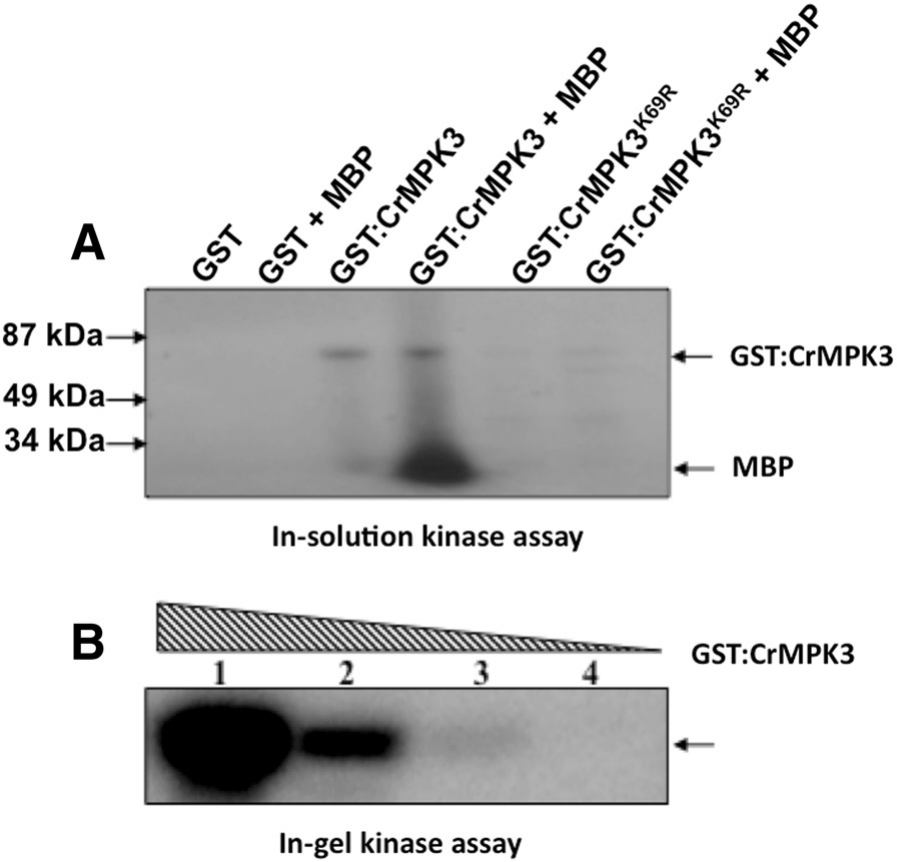
**CrMPK3 shows autophosphorylation as well as MBP phosphorylation.** (**A**) CrMPK3 expressed in *E. coli*, BL21 strain as GST fusion was analyzed for autophosphorylation, as well as phosphorylation of MBP in an *in-solution* kinase assay using radiolabled ATP. GST alone was used in the assay and showed no auto- and substrate phosphorylation. (**B**). An *in-gel* kinase assay with decreasing concentrations of GST-CrMPK3 using MBP as substrate. The amount of recombinant proteins used was 1 μg; 0.1 μg; 0.01 μg; 0.001 μg from lane1 to 4. The phosphorylated MBP was visualized by autoradiography. The experiments have been repeated three times with similar result.

To demonstrate that *CrMPK3* encodes a functional MAPK, its complete ORF was cloned into pGEX-4-T2 vector and transformed into protease deficient strain of *E. coli* BL21. The bacterially expressed glutathione S-transferase (GST):CrMPK3 fusion protein was affinity purified and used in an in-solution kinase assay alone as well as with myelin basic protein (MBP) as substrate. The recombinant protein showed auto-phosphorylation as well as phosphorylation of MBP (Figure 3A) suggesting that *CrMPK3* encodes an active MAP kinase. Kinase inactive form of CrMPK3 generated by mutating lysine (K) at 69th amino acid residue to arginine (R) in ATP binding site, CrMPK3^K69R^ failed either to auto phosphorylate or to phosphorylate MBP (Figure 3A). The CrMPK3 activity was further analyzed in an in-gel kinase assay with decreasing amount of recombinant protein in factor of 10 using MBP as substrate. As expected, the activity of purified fusion protein decreased with decrease in kinase concentration (Figure 3B). The inactive form, CrMPK3^K69R^ showed no activity in in-gel kinase assay (data not shown).

### CrMPK3 moves to nucleus upon wounding

**Figure 4:**
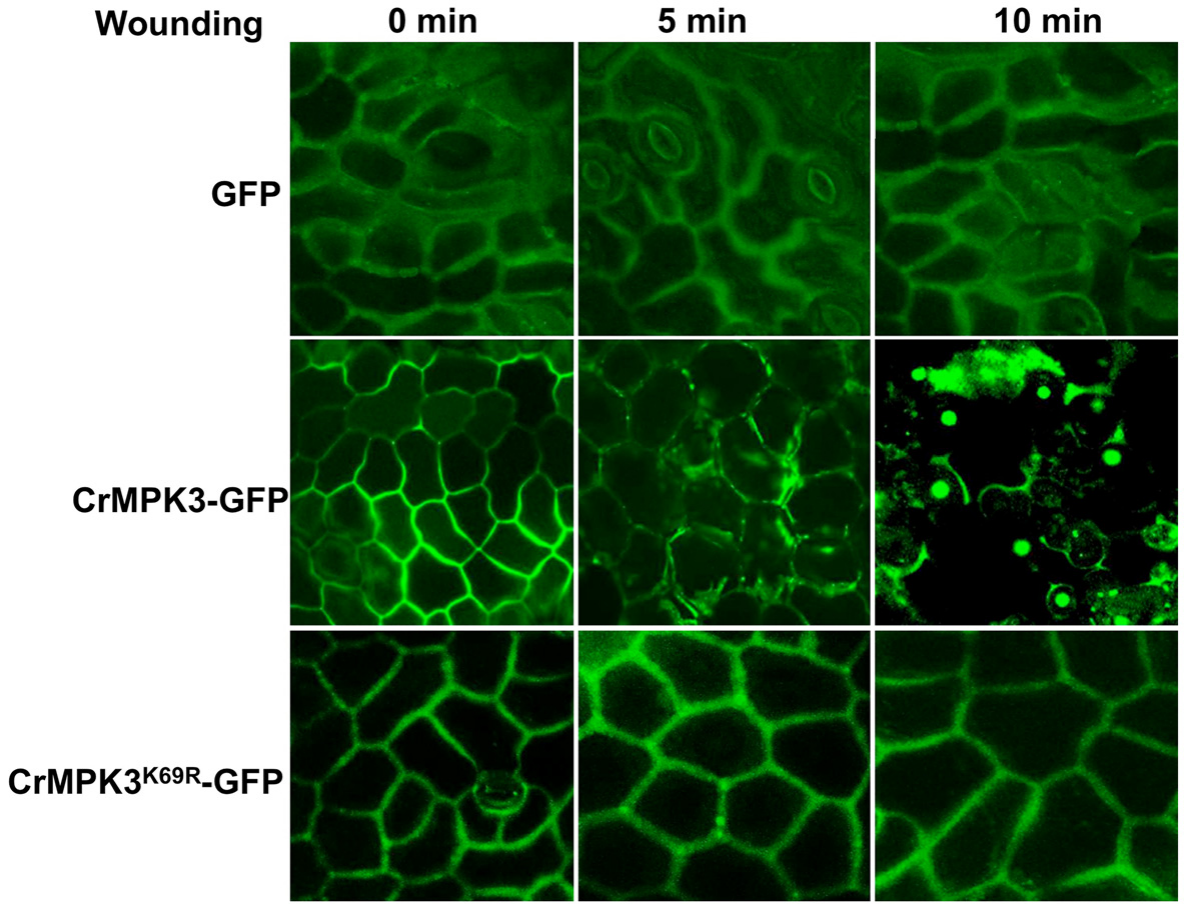
**CrMPK3 migrates towards nucleus upon wounding. ***C. roseus* leaf discs transiently transformed with CrMPK3-GFP/CrMPK3^K69R^ in pCAMBIA1303 vector or only pCAMBIA1303 vector and observed under confocal microscope without wounding and after wounding at the mentioned time points. The experiment was repeated twice with similar results.

To understand the fate of CrMPK3 in planta, subcellular localization of CrMPK3 was checked. For this, reading frame of *CrMPK3* was fused in frame with that of Green Fluorescent Protein (GFP) in pCAMBIA 1303 vector. *Agrobacterium tumefaciens* strain GV3101 transformed with this construct was used to transiently transform *C. roseus* leaf discs and viewed under confocal microscope after three days of incubation. CrMPK3 was found localized mostly inside the cell along plasma membrane and cytoplasm (Figure 4). Plasmolysis of the cells using mannitol have confirmed the fusion protein to be localized in plasma membrane and cytoplasm (Additional file 4). However, when the leaf tissue was wounded by cutting into smaller pieces and viewed under microscope after 5 min of wounding, there appeared a shift in GFP fluorescence dispersed mostly in cytoplasm (Figure 4). When the same slide was observed after ten minutes, most of the fluorescence was found localized to nucleus (Figure 4). Positions of the nucleus in slide are shown by DAPI staining which coincides to that of CrMPK3 nuclear signal (Additional file 5). However, neither the CrMPK3^K69R^-GFP nor GFP alone showed wounding induced migration to nucleus (Figure 4). This indicates that only the active CrMPK3 migrates to nuclei upon activation by wounding. This finding is consistent with earlier reports of translocation of MAPK from cytoplasm to nucleus in response to external stimuli [16]. 

### CrMPK3 shows wounding, UV and MeJA inducible expression and activity

**Figure 5:**
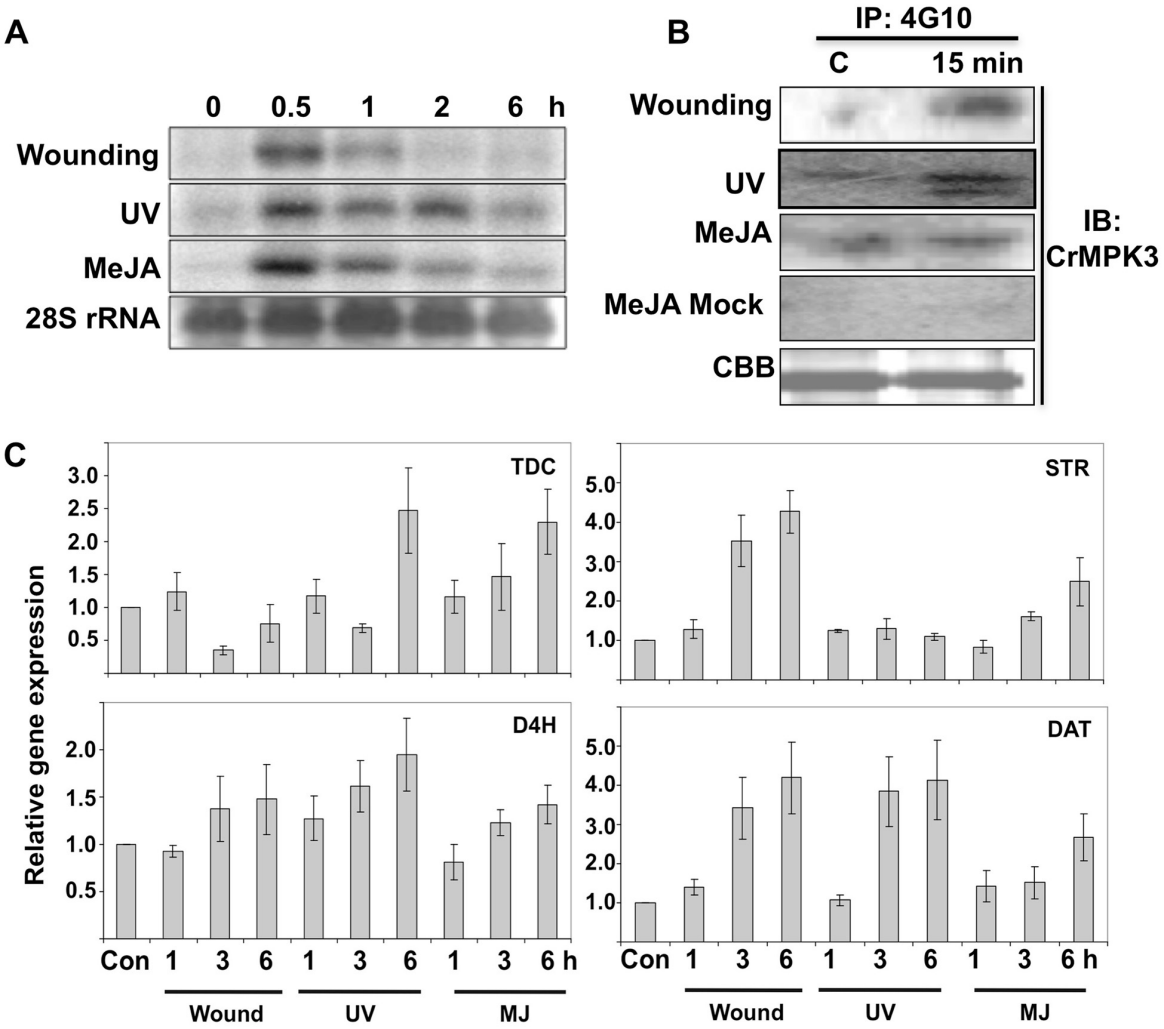
**Coregulation of *CrMPK3*expression, kinase activity and expression of MIA genes in *C. roseus*leaves upon wounding, UV, and MeJA treatment.** 6–8 weeks old *C. roseus* plants were subjected to wounding, UV treatment and MeJA application and leaves harvested at indicated time intervals. (**A**) Northern blot analysis of *CrMPK3.* Lowermost panel shows 28 S rRNA as loading control. (**B**) CrMPK3 activity assay in response to wounding, UV, MeJA treatments. Total protein extracts (200 μg) was subjected to immunoprecipitation with the 4 G10, phosphotyrosine antibody. The immunoprecipitated complex was electrophoresed on SDS 10%(w/v) polyacrylamide gel and immunoblot was performed using anti-CrMPK3. MeJA mock treatment was performed by applying solvent only (ethanol) in the similar fasion to that of MeJA. ‘CBB’ shows representative equal protein loading controls. (**C**) Quantitative RT-PCR analysis of key MIA pathway genes, *Tdc, Str, D4h* and *Dat*. Expression levels were normalized against expression of *C. roseus Actin* gene as an internal control and are shown relative to untreated control plants. The relative level of each gene in control plants at time 0 was standardized as 1. Values are presented as the mean and the errors bars indicate standard deviation of triplicate samples. The experiments were repeated three times with similar results.

Since tobacco WIPK and Arabidopsis MPK3, the close orthologs of CrMPK3 exhibit increase in both enzymatic activity and mRNA levels in response to various stimuli [17-19] we investigated whether *CrMPK3* gene is also induced by stress conditions that enhance MIA accumulation. Two-months-old *in vitro* grown *C. roseus* plants were subjected to different stress conditions like wounding and UV treatment as well as methyl jasmonate treatment. The transcript level of *CrMPK3* in response to these stresses was analyzed by northern blot analysis. A significant increase in the level of *CrMPK3* transcript was noted within 30 minutes of these treatments (Figure 5A). In case of wounding and MeJA treatments, the transcript level started to decline after 30 min of treatment. However, under UV treatment higher level of the *CrMPK3* transcript could be observed up to 2 hours post treatment before declining back to the basal level. 

To check whether stress inducible transcript accumulation of *CrMPK3* corroborates with its activity *in planta*, an immunoblot assay was performed using anti-CrMPK3 antibody. Leaves were subjected to wounding, UV stress treatment and methyl jasmonate application, the protein extracts prepared 15 minutes post treatments were immunoprecipitated with antiphosphotyrosine antibody 4 G10. The immunoprecipitates were subjected to western blot analysis with anti-CrMPK3 antibody. A distinct activation of 43 kDa MAP kinase is recognized by anti-CrMPK3 antibody in wounding, UV and MeJA treated tissues compared with control/mock treated sample (Figure 5B).

Since CrMPK3 and AtMPK3 show high sequence similarity, the commercially available AtMPK3 antibody (Sigma-aldrich) were used to assess activity of CrMPK3 in response to the stress conditions mentioned above in *C. roseus* leaves. The immunoprecipitation with the dually phosphorylated active form of the MAPK ERK1 (anti-pTEpY) antibody and immunoblot with αAtMPK3 showed the near similar results as were seen with CrMPK3 antibody (Additional file 6). Additionally, when immunoblot was perforemed using αCrMPK3 against total protein extracts of *Atmpk3* mutnat line and wild type plants (Col 0), the signals could be observed only in wild type plants (Additional file 7) validating the specificity of αCrMPK3 antibody.

Transcript up regulation and protein activation of CrMPK3 by wounding, UV treatment and methyl jasmonate prompted us to study key MIA pathway genes regulation by these stresses. *C. roseus* leaf tissue subjected to wounding, UV treatment and methyl jasmonate application was harvested at different time intervals post treatment. RNA was extracted from these tissues and quantitative-real time-PCR (qRT-PCR) was carried out for different genes of MIA pathway. The MIA genes analyzed were *Str* (Strictosidine synthase), *D4h* (Desacetoxyvindoline −4-hydroxylase), *Dat* (Deacetylvindoline 4-O-acetyltransferase) and *Tdc* (tryptophan decarboxylase) [20]. As shown in Figure 5C, all the treatments resulted in increase in expression of all the four MIA pathway genes. However only a slight increase in expression of *Str* is observed upon UV treatment.

### Transient over-expression of CrMPK3 in *C. roseus* leaves increases MIA pathway gene expression and alkaloid accumulation

**Figure 6:**
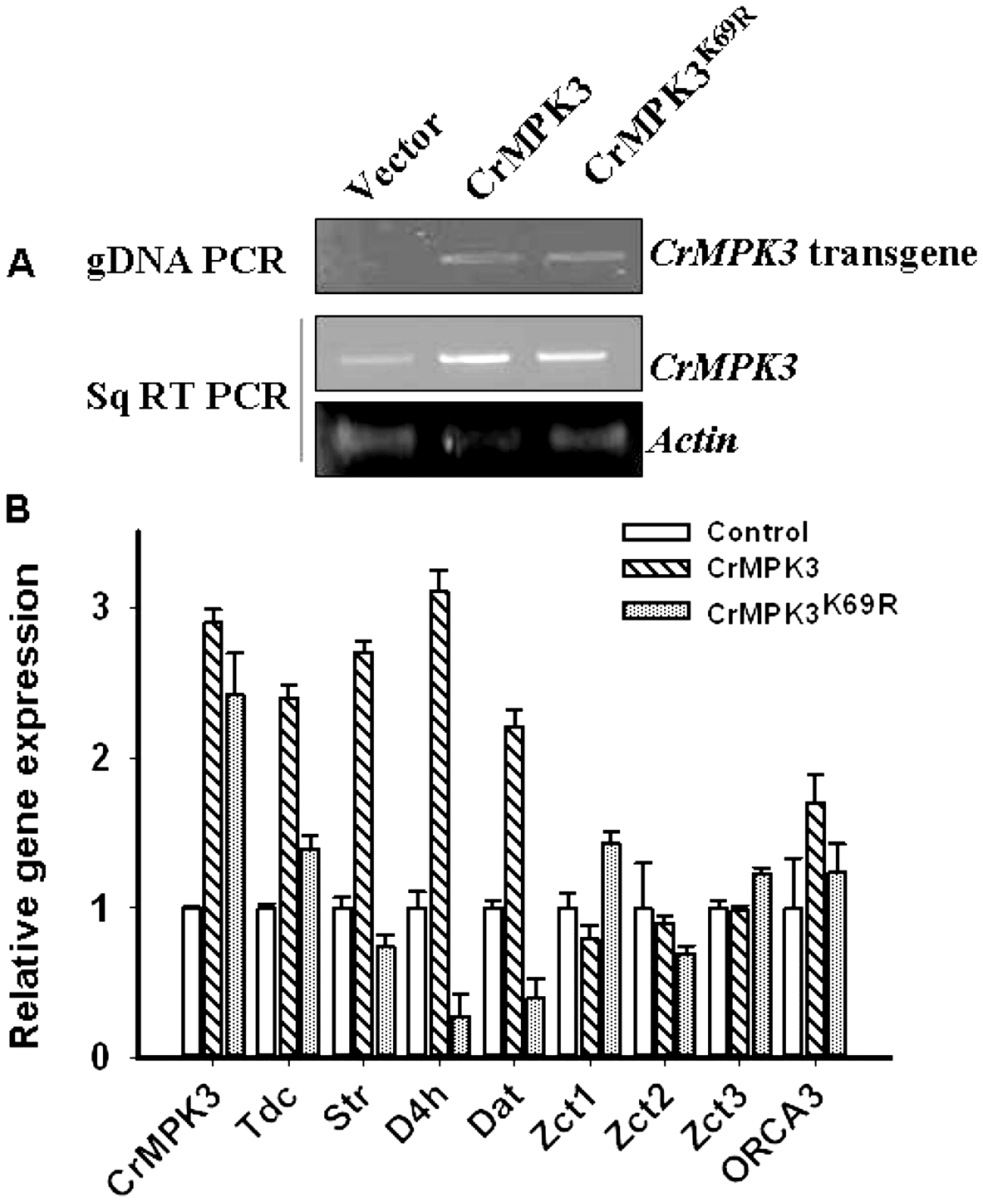
**Transient transformation of *CrMPK3*in*C. roseus*leaves increases expression of MIA pathway genes. ***C. roseus * leaves were transiently transformed with pCAMBIA 1303-*CrMPK3/CrMPK3 *^*K69R *^ construct or pCAMBIA 1303 (Empty Vector) alone by *A. tumifaciens* mediated transformation. Leaf samples were harvested 72 h post infiltration for RNA and protein extraction. **A**- PCR using genomic DNA of *CrMPK3/CrMPK.*^*3K69R*^ construct and vector transformed leaves (upper panel). A combination of vector and gene specific primers was used to check the presence of transgene. Middle panel shows expression of *CrMPK3* in *CrMPK3/CrMPK3*^*K69R*^ construct and vector transformed leaves by sqRT-PCR using gene specific primer pairs. Lower panel shows expressionof *Actin* gene as equal cDNA loading control. (**B**) Effect of *CrMPK3/CrMPK3*^*K69R*^ transient transformation on transcripts accumulation of key MIA pathway genes and regulators. qRT-PCR analysis was performed to check expression of *CrMPK3*, *Tdc, Str, D4H, Dat* and *Zct1, Zct2, Zct3 and Orca3.* Expression levels were normalized against expression of *C. roseus Actin* gene as an internal control and are shown relative to empty vector transformed leaf. The relative level of each gene in empty vector transformed leaf sample was standardized as 1. Values are presented as the mean and the errors bars indicate standard deviation of triplicate samples. The experiments were repeated three times with similar results.

The mRNA expression pattern of *CrMPK3* and key MIA pathway genes has shown a distinct co-regulation in response to different stress treatments. To draw a causal relationship between *CrMPK3* and MIA pathway genes and specific alkaloids, transient over-expression of *CrMPK3* and its kinase inactive version *CrMPK3*^*K69R*^ in *C. roseus* leaves were used. First we confirmed the presence of transgene in transiently transformed *C. roseus* leaves using genomic DNA PCR (Figure 6A). Expression analysis showed enhanced level of *CrMPK3* expression in *CrMPK3, CrMPK3*^*K69R*^ transformed leaves than that of control leaves (vector transformed) (Figure 6A, B). We then checked the expression of MIA pathway genes (*Tdc, Str*, *D4h*, *Dat*) and positive regulators *ORCA3* (Octadecanoid Responsive cathranthus APII domain factor 3) and repressors *ZCT1*, *2*, *3* (Z box binding factor 1, 2, 3) in the same samples using qRT-PCR analysis. The result obtained revealed a distinct up-regulation in the expression of *Tdc, Str*, *D4h*, *Dat* and *Orca3* and down regulation of *Zct1*, *Zct2* and *Zct3* in *CrMPK3* but not in CrMPK3^K69R^ transformed leaves (Figure 6B) compared to the empty vector transformed control leaves. In case of *CrMPK3*^*K69R*^ transformed leaves, inspite of high level of *CrMPK3* mRNA transcripts almost on par with CrMPK3, there were no major changes in transcripts level of MIA biosynthetic pathway genes (Figure 6B). The data obtained indicated that CrMPK3 is involved directly or indirectly in the up-regulation of MIA pathway genes and probably in more accumulation of the MIAs.

**Figure 7:**
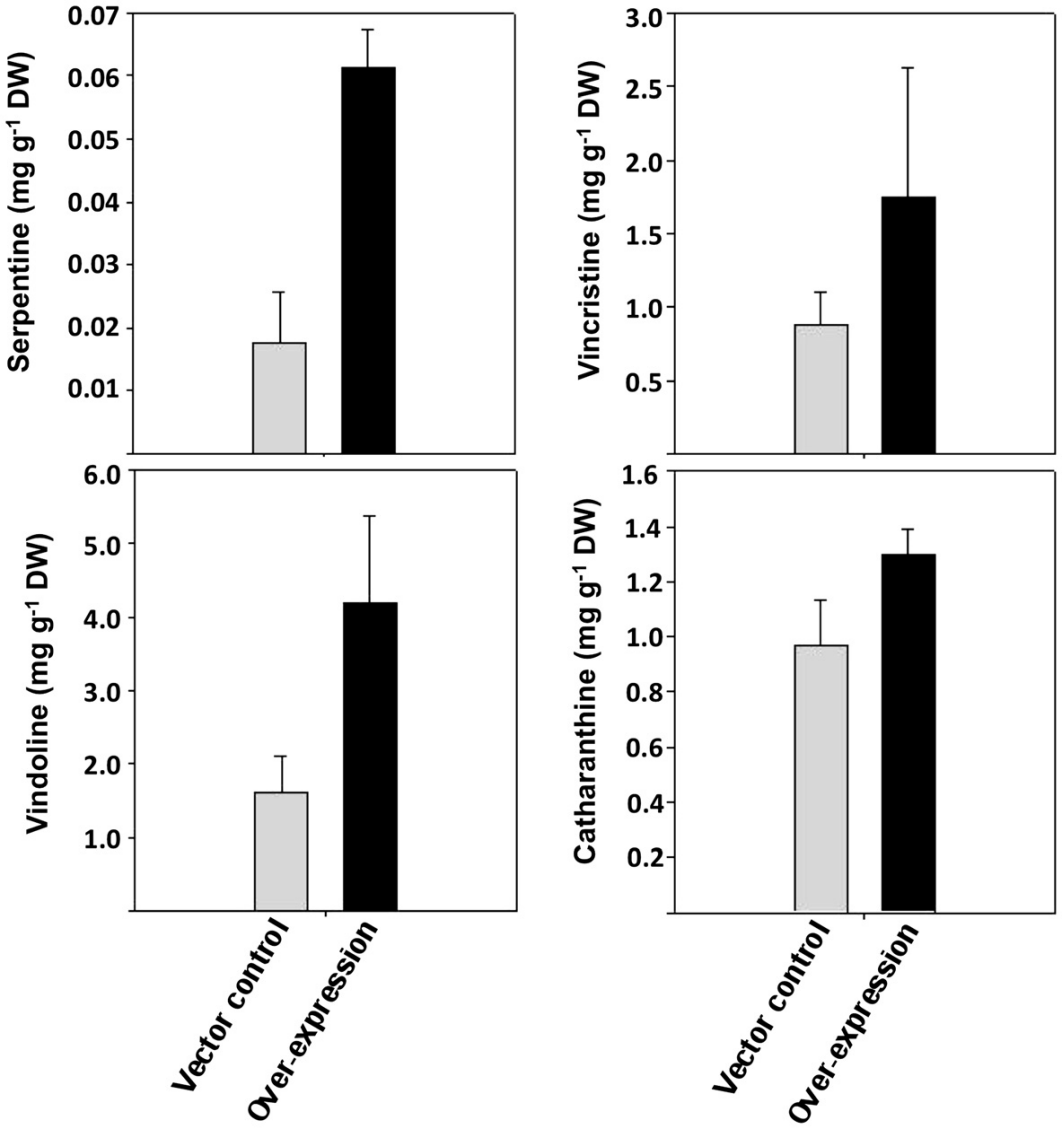
**Transient transformation of *CrMPK3 *in *C. roseus*leaves increases accumulation of specific MIAs.** Specific MIAs, serpentine, vindoline, vincristine and catharanthine accumulation in vector control or *CrMPK3* overexpressed leaves were measured. Values are presented as the mean and standard deviation of triplicate samples.

To get further insight into the role of CrMPK3 in stress regulated MIA accumulation we quantified some of the specific alkaloids in the transiently transformed *C. roseus* leaves. At least 8–10 leaves were used to quantify the alkaloids in both empty vector control and CrMPK3 transformed leaves. The estimation was carried out 72 hours post vacuum infiltration. Interestingly, the accumulation of serpentine, vincristine, vindoline and catharanthine were found to be more in the CrMPK3 transformed leaves (Figure 7). This result further supported the role of CrMPK3 in accumulation of MIAs.

## Discussion

*Catharanthus roseus* is one of the most pharmaceutically important plant and several efforts have been made to increase the alkaloid content of the plant. Some of them include, use of stress treatments such as wounding, UV treatment, heavy metal elicitation or elicitation by fungal cell wall components or treatment with hormones such as methyl jasmonate. These treatments lead to the increase in expression of MIA pathway genes and accumulation of alkaloids in higher amounts. JA responsive *Str* and *Tdc* expression is sensitive to protein kinase inhibitors [3] suggesting that phosphorylation plays a key role in the activation of MIA pathway genes. MAP kinases, being the important class of protein kinases involved in signal transduction are operative during biotic or abiotic stresses; its involvement in the elicitor mediated signal transduction leading to higher accumulation of alkaloids cannot be overruled. In this study, we report the isolation of a full-length mitogen activated protein kinase from *C. roseus, CrMPK3*. The deduced amino acid sequence showed 86% identity with WIPK from *Nicotiana benthamiana* and LeMPK3 from *Lycopersicon esculentum*, 84% identity with AtMPK3 and 78% identity with OsMPK6. Amino acid sequence analysis revealed the presence of 11 subdomains and a TEY motif between VII and VIII subdomains, typical of a MAP kinase. Further, *in solution* kinase assay of the GST-tagged CrMPK3 confirmed it to be an active MAP kinase.

Regulations of MAP kinases have been observed both at transcriptional level as well as posttranslational level [21,22]. Our findings with CrMPK3 have shown similar results. Enhanced transcript accumulation of *CrMPK3* was observed in response to wounding, UV exposure and MeJA treatments. The stress inducible expression of *CrMPK3* is in agreement with its orthologs in Arabidopsis (AtMPK3) and other plants where rapid expression was observed for MPK3 in response to stress [23,24]. Further in tomato, UV B specific activation of LeMPK3 has been reported [18]. It is important to note that CrMPK3 showed high homology with LeMPK3. These studies indicate conserved biological function of the close orthologs.

Further, wounding, UV and MeJA activated MAP kinases could be immunoprecipitated with 4 G10, a monoclonal anti-phosphotyrosine antibody [25,26] and identified with CrMPK3 antibody in an immunoblot analysis. The results confirmed that CrMPK3 is activated by wounding, UV and MeJA treatment. Interestingly, an activation of a putative MAPK has been shown in response to UV in *C. roseus* cell suspension culture (CSC) [27]. The UV-B responsive putative MAPK activity was found to play significant roles in stimulation of *Tdc* and *Str* genes and accumulation of catharanthine in response to UV. However, this specific MAPK has not been identified and gene encoding the same has not been cloned. Nevertheless, the MAPK activity shown earlier [27] is unlikely to be of CrMPK3 since its activity is observed at around 49 kDa where as that of CrMPK3 is of 43 kDa. Intriguingly our in gel kinase assay showed activation of more than one MAPK in response to UV. It is important to note that the study [27] has been conducted using *C. roseus* cell suspension culture (CSC) which lack cellular differentiation and provide an entirely different environmental conditions than naturally grown plants. Cellular differentiation has been considered one of the important factors for expression of genes of MIA and its accumulations [28-30]. The difference in activation of distinct MAPKs could be attributed to the different type of biological systems used for these studies. In agreement with this, distinct results with regard to CSC and mature plants have been observed in rice [31].

MAP kinase translocation into nucleus upon their activation is reported in many studies. The nuclear translocation of ERM (Elicitor responsive MAP) kinase in parsley within 10 minutes in response to elicitor treatment has been reported [16]. The nuclear translocation of CrMPK3 in response to wounding in *C. roseus* is in agreement with earlier report [16]. MAP kinases are widely speculated to directly or indirectly interact with transcription factors in the nucleus for gene regulation. The transfer of activated MAP kinase upon wounding to nucleus in the present study supports the function of MAPKs.

Since biosynthesis of MIAs is responsive to environmental signals, transcript accumulation of a few MIA pathway genes in response to wounding, UV treatment and Methyl jasmonate application was studied. A distinct regulation of the MIA pathway genes was observed at transcript level. Since *CrMPK3* transcript also showed regulation in response to these treatments, it was thought worthwhile to study the effect of transient over-expression of *CrMPK3* on MIA pathway genes. *C. roseus* leaves were transiently transformed with *CrMPK3* binary construct by vacuum infiltration method and transiently transformed leaves were studied for the expression of MIA pathway genes. An increase in CrMPK3 activity was observed in the transiently transformed leaves. The transient transformed leaves also showed a distinct up-regulation in transcription of *TdcDat* and *D4h.* Transcript regulation of the positive and negative regulators of MIA pathway genes was also studied. Interestingly, *Orca3,* a positive regulator of the pathway showed up-regulation, while *Zct1Zct2* and *Zct3* the repressors of MIA pathway [32,33], showed down regulation. Zcts are members of zinc finger type transcription factor family and are known repressor of MIA biosynthesis pathway genes [32]. *Orca3* is a member of AP2 transcription factor and are known positive regulator of MIA pathway genes [33]. Moreover, the increased transcripts of MIA genes also resulted in higher accumulation of specific alkaloids in transformed leaf samples. A positive correlation has been reported between up-regulation of *Dat* and *D4h* transcripts and accumulation of related MIAs *in C. roseus*[34]. The up-regulation in the transcript level of *Tdc*, Str, *Dat* and *D4h* can be partly explained by upregulation of the positive regulator and down-regulation of the repressors. The enhanced transcripts level of key genes of MIA as well as increased accumulation of the specific alkaloids in transiently transformed leaves suggest a positive effect of CrMPK3 on MIA biosynthesis. Role of the members of MAPKs in regulating defence compounds such as phytoalexins have been well documented in Arabidopsis and rice. In Arabidopsis MPK3 and MPK6 have been shown to regulate multiple genes in ethylene and camalexin biosynthesis [5,35]. Similarly in rice MKK4-MPK3/MPK6 cascade regulate expression of several genes of phytoalexin biosynthesis and accumulation of phytoalexins in response to MAMP [36].

Our present study reports, a MAP kinase from a medicinally important, *C. roseus* and its involvement, directly or indirectly in regulating the synthesis of monoterpenoid indole alkaloids. Based on these findings it appears that CrMPK3 could be a potential candidate that can be engineered for better production of specific alkaloid in *C. roseus*.

## Conclusions

In the present study, cloning of the first MAPK, *CrMPK3* from medicinally important plant *Catharanthus roseus* has been reported. The *CrMPK3* encodes an active MAP kinase with 372 amino acids and molecular weight ~43 kDa. In-silico analysis of amino acid sequence of CrMPK3 shows that it possesses all the basic features of MAP kinase with ‘TEY’ phosphorylation motif. Phylogenetic analysis of CrMPK3 with closely related MAPKs suggests that it belongs to group A MAPK [14] as it shows high sequence identity with tobacco WIPK and Arabidopsis AtMPK3. Interestingly, *CrMPK3* shows UV, wounding and MeJA inducible expression pattern that is in harmony with the expression pattern of many of the key genes and regulators of MIA biosynthetic pathway and MIA accumulations (Figure 5) [33,37]. Enhanced expression of *CrMPK3* in the above conditions also reflected in its increased kinase activity in all studied conditions. Role of CrMPK3 in wounding is further substantiated with wounding induced nuclear localization of CrMPK3-GFP, which is otherwise localized in cytoplasm (Figure 4). Further concordance of CrMPK3 and MIAs comes from the transient expression of *CrMPK3* in *C. roseus* leaves, which not only resulted in increased expression of master regulator, *Orca3* and key genes of MIA pathway but also more accumulation of important alkaloids such as serpentine, vindoline, vincristine and catharanthine (Figure 6,7). From these studies we infer that CrMPK3 may form an important component of signal transduction pathway leading to stress induced accumulation of MIAs in *C. roseus*. Future studies with stable transgenics of *CrMPK3* and identification of interacting partner of CrMPK3 in the form of the already identified MIA pathway regulators could shed more light on signal transduction network. Nonetheless, the present work is a miniature step towards unraveling the complex signal transduction mechanism in this model medicinal plant.

## Methods

### Plant material and treatments

Two months old *Catharanthus roseus* var. Nirmal plants growing in greenhouse at a temperature of 28°C were subjected to various stress treatments. For wounding intact leaves of *C. roseus* plants were damaged upto 40%of their leaf lamina using a surgical blade. The leaf samples were harvested at different time points mentioned in the legend of respective figures by snap freezing in liquid N_2_ and stored in −80°C for further analysis.

UV treatment was given by exposing plants under UV C (253 μmol m^-2^ s^-1^) lamp for two minutes. Methyl jasmonate treatment was given by painting the leaves of *C. roseus* with Methyl jasmonate (50 μM) using a cotton ball dipped in methyl jasmonate solution.

### Isolation of *CrMPK3*

A partial cDNA clone of *C. roseus* MAPK (438 bp) was amplified using the specific primers, MAPKF1: 5′-CTCAAGCTTCTTCGTCATAT-3′ (forward) and MAPKR1: 5′-GACAGACCACACATCAATTG-3′ (reverse) designed from *CrMAPK* EST (GenBank accession No. AJ537469). Amplification was carried out at 94°C for 30 sec, 52°C for 30 sec, and 72°C for 30 sec for 30 cycles using *Taq* DNA polymerase (Bangalore Genei, India). The partial *CrMAPK* was used as a probe to screen a λ-ZapII oriented YE elicited *C. roseus* leaf-specific cDNA library to get full length clone. Recombinant plaques were screened by plaque hybridization method. Positive plaque obtained after tertiary screening led to identification of a 630 bp long partial *CrMAPK* clone with missing 5′ end. The 5′ end of the gene was successfully amplified using a combination of 5′ degenerate primer and reverse specific primer using *C. roseus* cDNA as template. The primer sequences were as follows: 5′-ATGGYTGATGCWAATATGGGTG-3′ (forward) and 5′-TTATGCATATTCTGGATTTAGAGCC-3′ (reverse). The amplified fragment was cloned into pGEM-T easy cloning vector (Promega), sequenced. Full length *CrMAPK* clone was identified using ORF finder tool (NCBI). The full length cDNA of *C. roseus* MAPK was PCR amplified from *C. roseus* cDNA using gene specific primer pair (FLMPKF1: 5′-ATGGTTGATGCAAATATGG-3′ and FLMPKR1: 5′-TTATGCATATTCTGGATT-3′) and cloned in PGEM-T Easy vector (Promega) and sequenced.

### Preparation of GST fusion proteins

Full length *CrMPK3* was cloned in expression vector pGEX-4 T-2 at *Bam*HI and *Xho*I restriction sites. GST-CrMPK3 fusion protein was induced with 1 mM isopropyl thio-β-D-galactosidase (IPTG) in *E. coli* strain BL-21. The fusion protein was purified using glutathione beads (GE Healthcare) following manufacturer’s instructions.

### Generation of Kinase inactive GST-CrMPK3 fusion protein

Kinase inactive GST:CrMPK3 fusion protein was generated by mutating a conserved lysine (K) at 69^th^ residue in the ATP-binding domain of CrMPK3 to arginine (R). The directed mutagenesis was carried out using *CrMPK3* in pGEX-4 T-2 as template by mutagenic primer pair (5′-ATGGTGGCGATTAGGAAAATAGCT-3′ and 5′-AGCTATTTTCCTAATCGCCACCAT-3′). The PCR amplification was carried out at 94°C for 30 sec, 52°C for 30 sec, and 72°C for 5 min for 30 cycles followed by final extension at 72°C for 10 min. PCR product was digested overnight with *Dpn*I and the digested product was transformed in protease deficient *E. coli* strain BL-21 (DE3). The point mutation K69R was verified by sequencing. The purified GST:CrMPK3^K69R^ was obtained following the same protocol as mentioned before.

### In-gel kinase assay

The in-gel kinase activity was carried out as described previously [26]. 20 μg of total protein was fractionated on a 10% polyacrylamide gel containing 0.1%SDS and 0.5 mg/ ml bovine brain myelin basic protein (MBP) (Sigma Aldrich). After electrophoresis, the SDS from the gel was removed with buffer (25 mM Tris–HCl pH 7.5, 0.5 mM DTT, 5 mM Na_3_VO_4,_ 0.1 mM NaF, 0.5 mg ml^-1^ BSA, 0.1%Triton X 100) followed by renaturation in buffer (25 mM Tris–HCl pH 7.5, 0.5 mM DTT, 5 mM Na_3_VO_4_, 0.1 mM NaF) at 4°C overnight. MBP phosphorylation was performed by incubating the gel in 20 ml of reaction buffer (25 mM Tris HCl pH 7.5, 2 mM EGTA, 12 mM MgCl_2_, 1 mM DTT, 0.1 mM Na_3_VO_4_, 1 μM ATP and 50 μCi of γ32P-ATP (3000 Ci mmol^-1^) for 60 min at room temperature. The gel was washed three times with 5%TCA and 1%Sodium pyrophosphate and dried on filter paper and autoradiographed in phosphorimager (Typhoon, GE health care).

### In solution kinase assay

GST fusion protein (5 μg) was incubated with 5 μg of MBP and 1 μCi of γ-^32^P-labelled ATP in a 15 μl reaction mixture (25 mM Tris-Cl pH 7.5, 5 mM MgCl_2_, 25 μM ATP, 1 mM EGTA and 1 mM DTT) at 30°C for 20 minutes. The reaction was stopped with 5× SDS loading buffer and the samples were resolved on a 15% polyacrylamide gel. The gel was dried on Whatman 3MM paper and exposed to X-ray film.

### Protein extraction and immunoblot analysis

Frozen leaf tissue was ground in liquid nitrogen and homogenized in 1 ml of ice-cold extraction buffer (100 mM HEPES -KOH pH 7.5, 5 mM EDTA, 5 mM EGTA, 10 mM DTT, 10 mM Na_3_VO_4_, 10 mM NaF, 50 mM β-glycerol phosphate, 1 mM PMSF, 10%glycerol and 7.5%PVPP). Extracts were centrifuged and the clear supernatant was recovered. For immunoprecipitation 200 μg of extracted crude protein was incubated with 1 μg of 4 G10 antiphosphotyrosine monoclonal antibody (Upstate Biotechnology, NY)/ dually phosphorylated active form of the MAPK ERK1, pTEpY antibody (Cell Signalling Technology) in 250 μl volume of extraction buffer (without PVPP) with 250 mM NaCl and 0.1%(v/v) Nonidet P40. The assay was shaken at 4°C for 2 h and after the subsequent addition of 30 μl of protein A-Sepharose (GE Healthcare) for overnight, followed by centrifugation (13,000 rpm) for 3 min at 4°C. The beads were washed thrice with extraction buffer, and boiled for 5 min in 30 μl of 5x loading buffer (0.625 M Tris–HCl pH 6.8, 5%SDS, 50%glycerol, 0.125%bromophenol blue and DTT). The reaction products were used for protein gel blot analysis as described previously [38]. CrMPK3 polyclonal antibody was custom synthesized from Gene Care, India. Briefly, purified recombinant CrMPK3 was provided for raising polyclonal antibody in rabbit. The preimmune serum and serum after inoculation were collected and tested for their ability to bind the *C. roseus* proteins in immumoblot analysis. The preimmune serum failed to detect any protein band specific to *C. roseus* in immunoblot analysis (data not shown) A primary antibody dilution of 1:2000 was used for anti-CrMPK3.

### Generation of construct for sub-cellular localization study and transient transformation of *C. roseus*

Full length *CrMPK3* and *CrMPK3*^*K69R*^ was amplified with primers containing *Nco*I and *Spe*I restriction sites in the forward and reverse primers respectively. For sub-cellular localization construct, reverse primer lacked the stop codon so that coding region of *CrMPK3* is in frame with reading frame of green fluorescent protein however for transient transformation construct stop codon was included in reverse primer. Respective amplicons were cloned in pGEM-T Easy vector and confirmed by sequencing. The constructs were generated by digesting *CrMPK3* in pGEMT Easy vector with *Nco*I/ *Spe*I and inserting the excised fragment into the corresponding sites of the plant binary vector pCAMBIA 1303 digested with same set of restriction enzymes. The constructs were further confirmed by sequencing.

### RNA extraction and Northern Blot Analysis

RNA extraction and Northern blot analysis were performed as described earlier [39]. 20 μg RNA samples were separated on 1.2%denaturing agarose gel and blotted onto Hybond-N 14 membrane (GE, Piscataway, NJ, USA) using standard procedures. Prehybridization and hybridization of membranes were carried out at 60°C in modified church buffer (7%SDS, 0.5 M NaPO4, 10 mM EDTA, pH 7.2). Blots were hybridized with α-^32^P-dCTP-labeled full length cDNA clone of *CrMPK3*. Blots were washed in 2XSSC and 0.1%(w/v) SDS at room temperature for 10 min followed by washing in 0.5X SSC and 0.1%(w/v) SDS wash buffer at 60°C for 10 min. Membranes were exposed to Kodak X-OMAT film and signals were analyzed.

### Quantitative real time PCR (qRT-PCR) analysis

Total RNA was extracted from the infiltrated leaves by LiCl method [39] and treated with 10 units of RNase free DNase 1 (Takara Bio Inc., Japan) to remove genomic DNA contamination. Five micrograms of total RNA was subjected to first strand synthesis using Power Script Reverse Transcriptase according to the manufacturer’s procedure using oligo (dT) primer. qRT-PCR was conducted as have been described previously [20,40]. PCR amplification was conducted on one-tenth of the reaction. Primer pairs used for qRT-PCR analysis of different genes is mentioned in Additional file 8: Table 1.

### Transient transformation of *C. roseus* leaves by Vacuum infiltration

*CrMPK3/CrMPK3*^*K69R*^ in binary construct were used for transformation of competent cells of *Agrobacterium tumefaciens* strain GV3101. Six young leaves of *C. roseus* were submerged into a suspension of recombinant *A. tumefaciens* (OD_600_ =2) in MMA (0.43%[w/v] Murashige and Skoog basic salt mixture [Sigma, Deisenhofen, Germany], 10 mM MES [pH 5.6], 2%[w/v] sucrose, 200 μM acetosyringone) and incubated for 30 min under negative pressure generated by a vacuum pump. Leaves infiltrated with a suspension of empty vector were used as negative controls. Following infiltration, the leaves were briefly washed in tap water and dried on paper tissues to remove excess liquid. The leaves were placed on wet Whatman paper in plastic trays, sealed, and incubated for 60 h in a phytochamber at 25°C, with a 16 h photoperiod.

### Total alkaloid extraction and HPLC analysis

Alkaloid extraction and HPLC analysis was performed as mentioned elsewhere [34]. Briefly, total alkaloids were extracted from the dried leaf samples following the protocol described earlier [41]. Chromatographic separation of major indole alkaloids of *C. roseus* was carried out on a reversed-phase C18 column with binary gradient mobile phase profile [42]. Identification of the compounds were based on the retention time and comparison of the UV spectra with those of the authentic standards. Solutions of different authentic samples (catharanthine, vindoline, vinblastine, and serpentine) were prepared in methanol (0.25 mg ml^-1^) and used at different concentrations for the preparation of calibration graphs, linear in the range of 0.25–25 μg. Quantification analysis was repeated for three replicates of each tissue in parallel, and the means and standard deviations were calculated.

## Abbreviations

GFP: Green fluorescent protein; MeJA: Methyl jasmonate; MAPK: Mitogen activated protein kinase; MAPKK: Mitogen activated protein kinase kinase; MAPKKK: Mitogen activated protein kinase kinase kinase; MBP: Mylein basic protein; MIA: Monoterpenoid indole alkaloid; UV: Ultraviolet.

## Competing interests

The authors declare that they have no competing interest.

## Authors’ contributions

SKR performed most of the experiments and drafted the manuscript, DPW conducted IP and in-gel kinase assays and helped in drafting the manuscript, MJ and SKJ helped in quantitative real time PCR (qRT-PCR) analysis, PS helped in constructs preparations, confocal microscopy and raising Arabidopsis mutant plants, AHS helped in confocal microscopy work, BR helped in western blot analysis. AKS conceived the idea, designed and coordinated the experiments and drafted the manuscript. All authors read and approved the final manuscript.

## Supplementary Material

Additional file 1 **Activation of MAP Kinases by MeJA treatment in *C. roseus.*** 6*–*8 weeks old *C. roseus* plants were subjected to MeJA treatment at the mentioned concentrations. Control plant was given the mock treatment (Ethanol) in the same fashion as that of MeJA treatment. Activity of MAP kinase was assessed by in-gel kinase assay using MBP as a substrate polymerized in SDS 10%(w/v) polyacrylamide gel. Click here for file

Additional file 2 **Phylogenetic relationship of CrMPK3 to other plant MAPKs.** The amino acid sequence of the proteins used for generating phylogenetic tree is listed in GenBank database under the following accession numbers: OsMPK3 (A2XFC8), OsMPK6 (ABO69383), NtF4 (X83880), SIMK (X66469), PsD5 (X70703), AtMPK6 (D21842), ZmMPK5 (AB016802), WIPK (D61377), AtMPK3 (D21839), ZmMPK4 (AB016801), AsMAP1 (X79993), LeMPK3 (AAP20421 ) and StMAPK (BAE44363). Click here for file

Additional file 3 **Deduced amino acid sequence of CrMPK3.** Amino acid sequence of CrMPK3 highlighting different subdomains of MAP kinase. Conserved TEY motif present between subdomain VII and VIII is underlined. Click here for file

Additional file 4 **Plasmolysis of *C. roseus*leaf cells to confirm the subcellular localization of CrMPK3-GFP fusion protein.** Plasmolysis was achieved by incubating the cells in 1 M mannitol solution for 2 hours. Click here for file

Additional file 5 **Nuclear localization of CrMPK3-GFP upon wounding. ***C. roseus * leaf discs transiently transformed with CrMPK3-GFP cloned in pCAMBIA1303 vector and observed under confocal microscope 10 min after wounding. **A-B**: CrMPK3-GFP fluorescence and bright field respectively. **C**: DAPI staining shows position of nucleus. Click here for file

Additional file 6 **CrMPK3 activation in response to wounding, UV, MeJA treatments.** Total protein extracts (200 μg) was subjected to immunoprecipitation with the pTEpY antibody. The immunoprecipitated complex was electrophoresed on SDS 10%(w/v) polyacrylamide gel and immunoblot was performed using anti-AtMPK3. MeJA mock treatment was performed by applying solvent only (ethanol) in the similar fasion to that of MeJA. Representative CBB stained total protein show equal loading control. Click here for file

Additional file 7 **Specificity of CrMPK3 antibody.** Immunoblot was performed using CrMPK3 antibody against total protein extract of Arabidopsis *Atmpk3* mutant line and wild type plants (Col 0). Click here for file

Additional file 8 List of genes and primer pairs for Q RT-PCR. Click here for file
