# Supplementary figures and images for: CrMPK3, a mitogen activated protein kinase from Catharanthus roseus and its possible role in stress induced biosynthesis of monoterpenoid indole alkaloids
Source: BMC Plant Biol. 2012 Aug 7;12:134. doi: 10.1186/1471-2229-12-134 (PMC3487899; doi:10.1186/1471-2229-12-134)

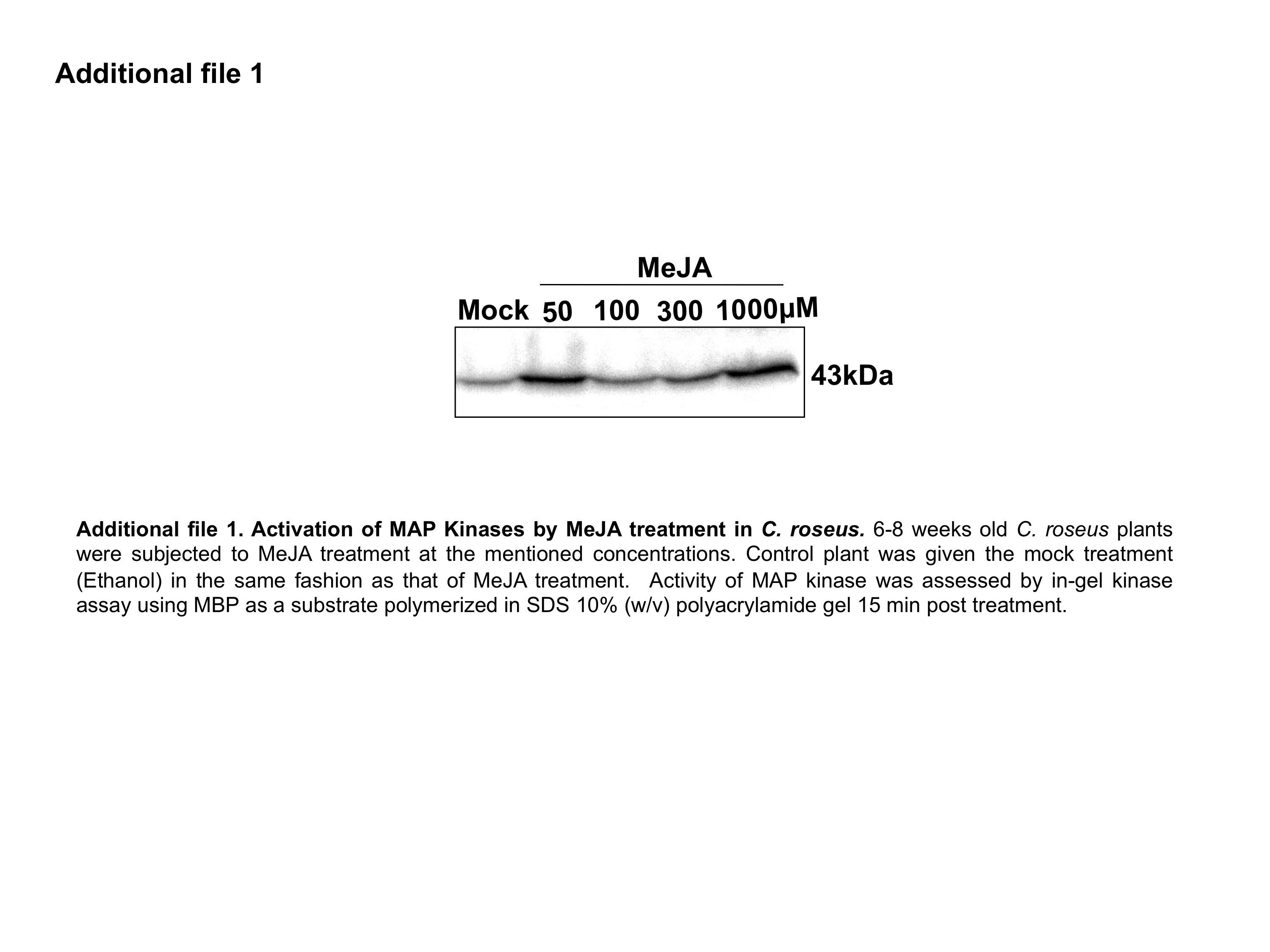

Supplement: Additional file 1 — Activation of MAP Kinases by MeJA treatment in C. roseus. 6–8 weeks old C. roseus plants were subjected to MeJA treatment at the mentioned concentrations. Control plant was given the mock treatment (Ethanol) in the same fashion as that of MeJA treatment. Activity of MAP kinase was assessed by in-gel kinase assay using MBP as a substrate polymerized in SDS 10%(w/v) polyacrylamide gel. [file 1471-2229-12-134-S1.jpeg]

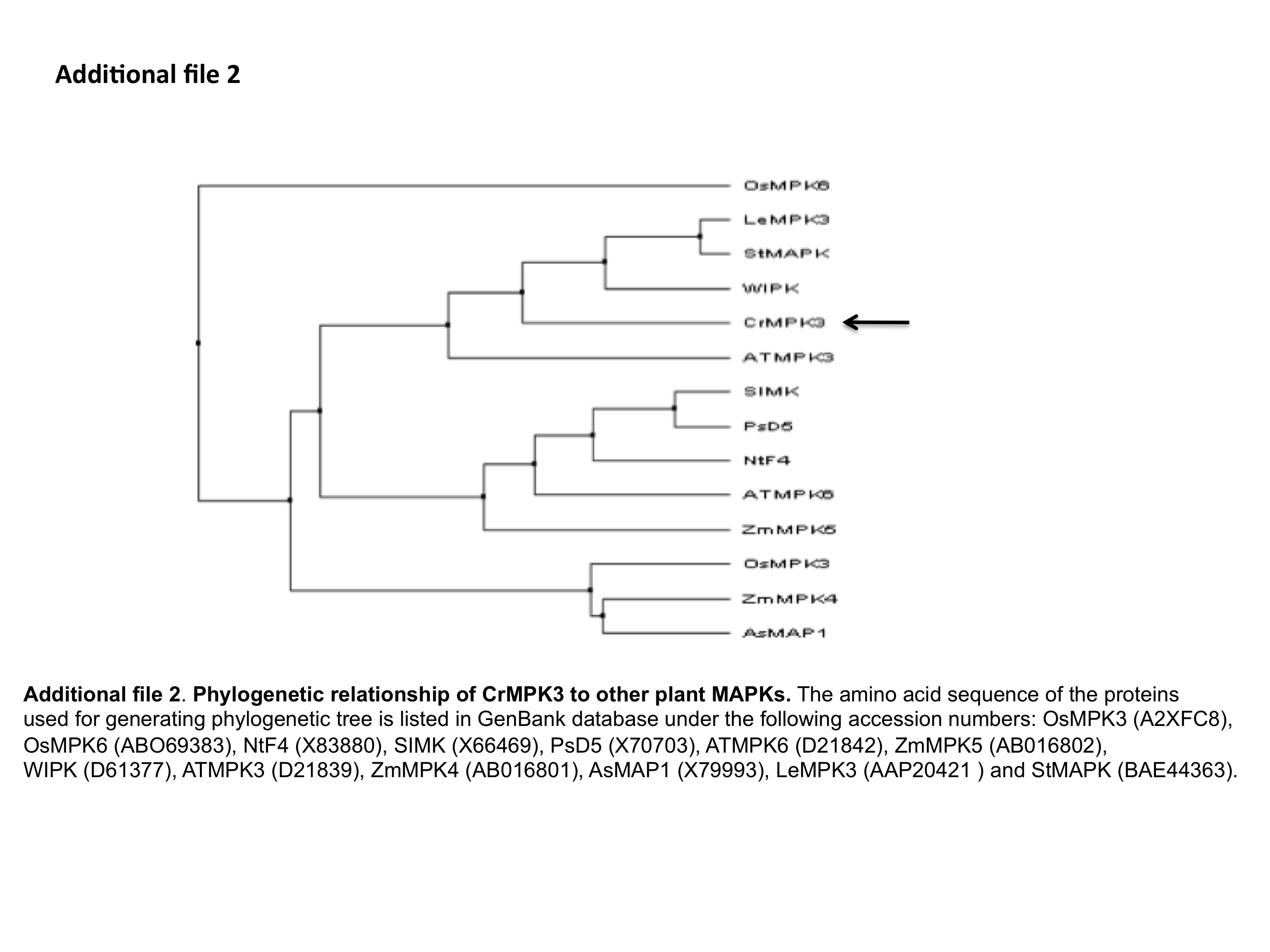

Supplement: Additional file 2 — Phylogenetic relationship of CrMPK3 to other plant MAPKs. The amino acid sequence of the proteins used for generating phylogenetic tree is listed in GenBank database under the following accession numbers: OsMPK3 (A2XFC8), OsMPK6 (ABO69383), NtF4 (X83880), SIMK (X66469), PsD5 (X70703), AtMPK6 (D21842), ZmMPK5 (AB016802), WIPK (D61377), AtMPK3 (D21839), ZmMPK4 (AB016801), AsMAP1 (X79993), LeMPK3 (AAP20421 ) and StMAPK (BAE44363). [file 1471-2229-12-134-S2.jpeg]

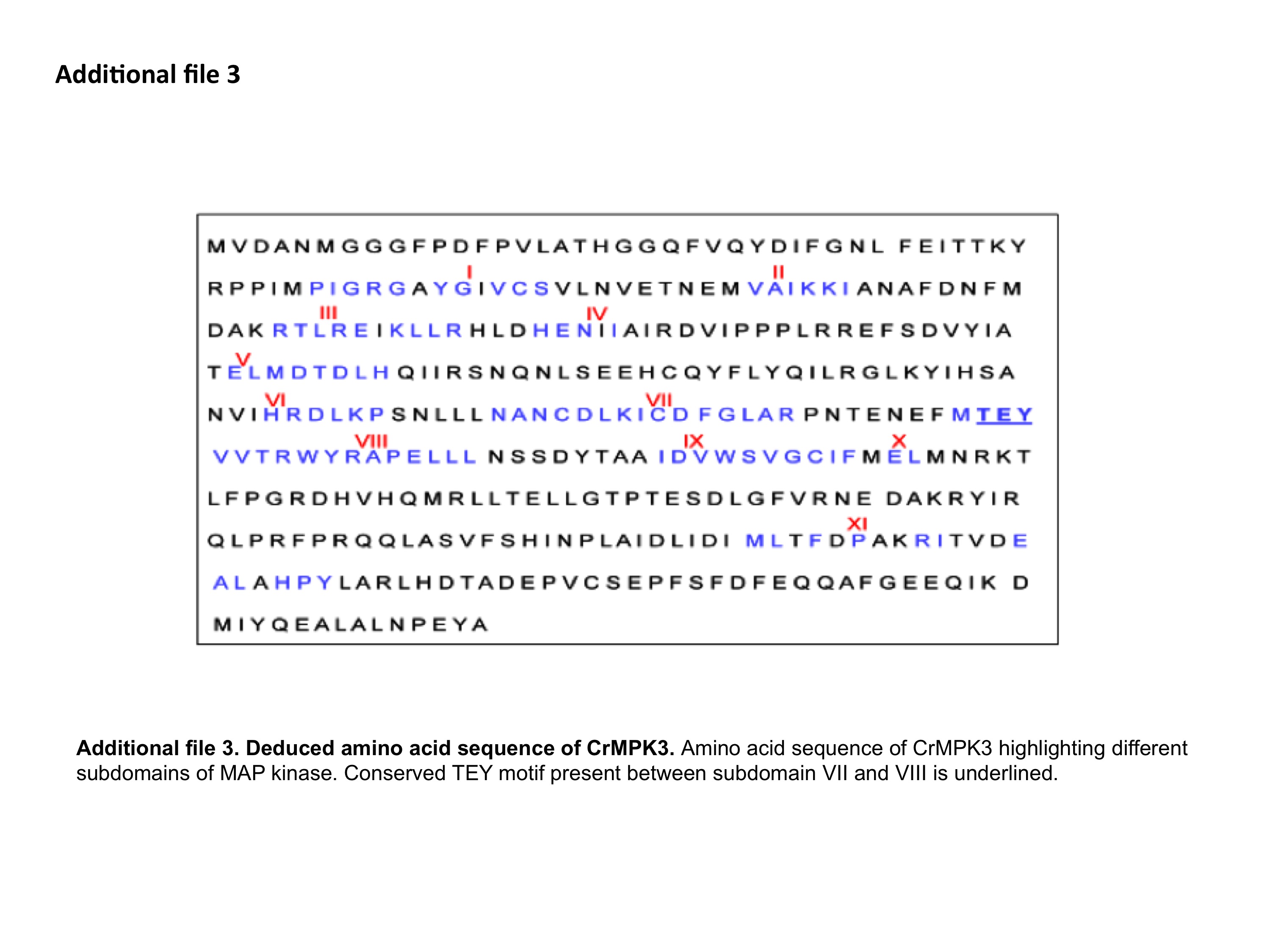

Supplement: Additional file 3 — Deduced amino acid sequence of CrMPK3. Amino acid sequence of CrMPK3 highlighting different subdomains of MAP kinase. Conserved TEY motif present between subdomain VII and VIII is underlined. [file 1471-2229-12-134-S3.jpeg]

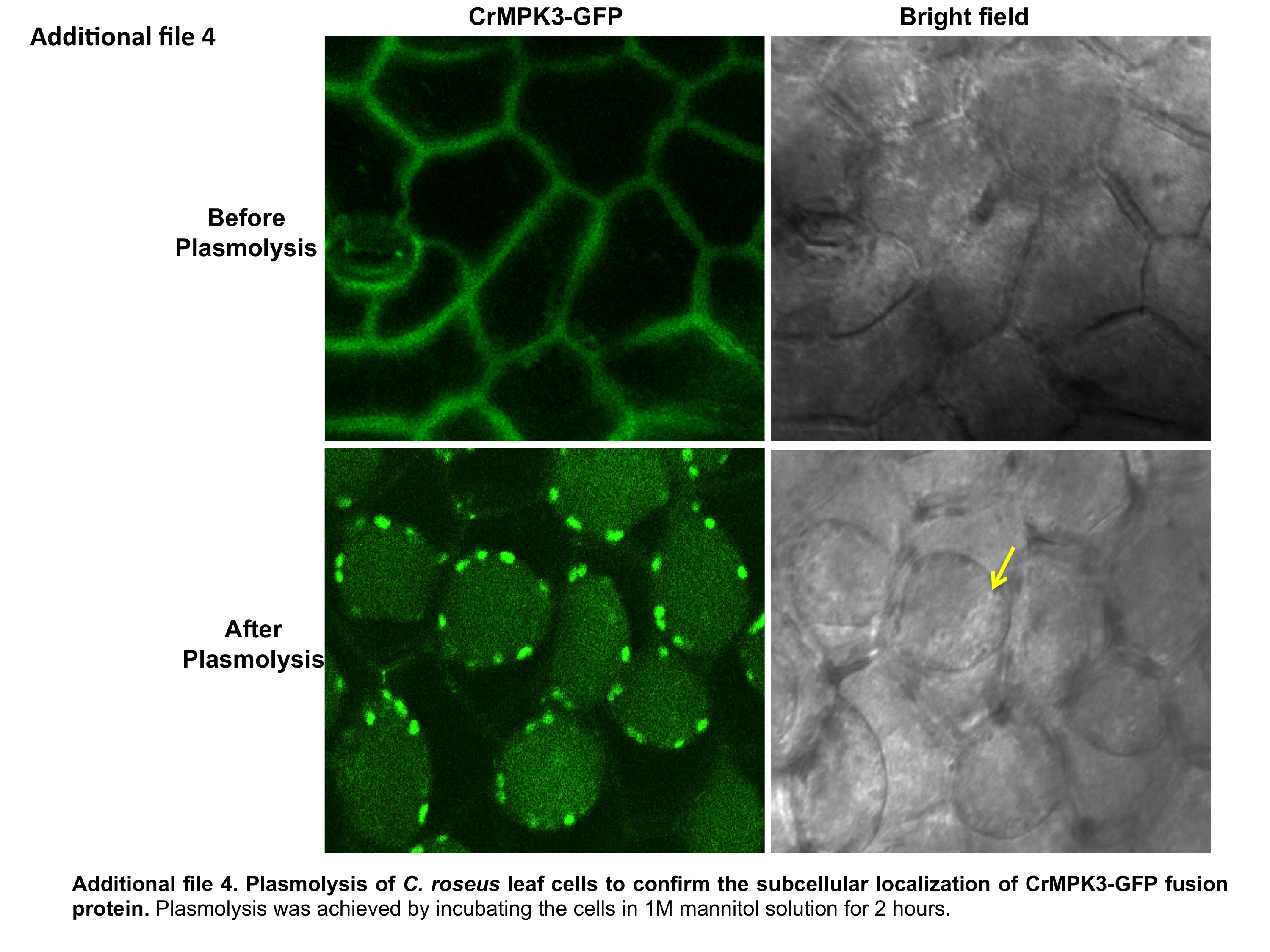

Supplement: Additional file 4 — Plasmolysis of C. roseusleaf cells to confirm the subcellular localization of CrMPK3-GFP fusion protein. Plasmolysis was achieved by incubating the cells in 1 M mannitol solution for 2 hours. [file 1471-2229-12-134-S4.jpeg]

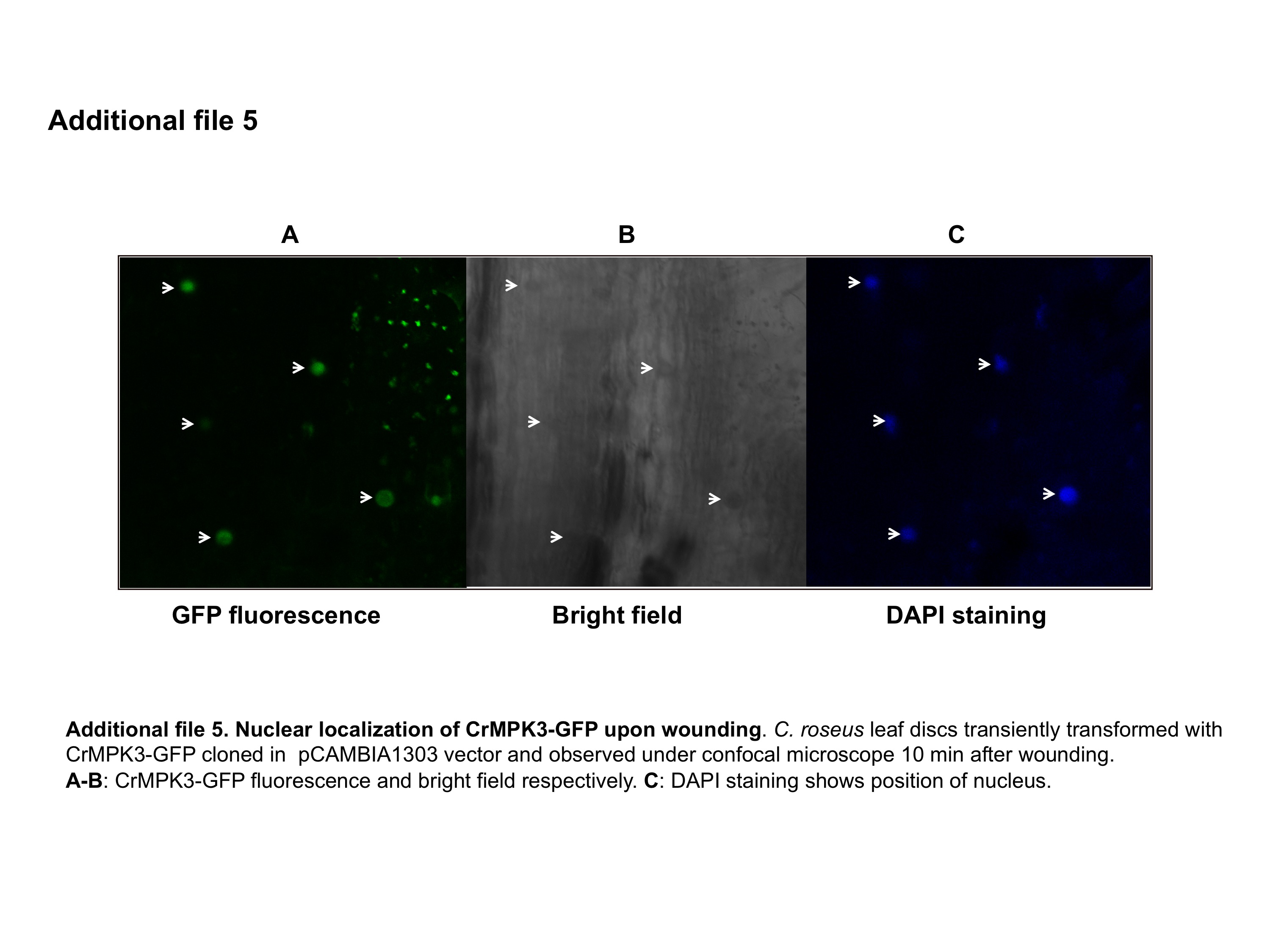

Supplement: Additional file 5 — Nuclear localization of CrMPK3-GFP upon wounding. C. roseus leaf discs transiently transformed with CrMPK3-GFP cloned in pCAMBIA1303 vector and observed under confocal microscope 10 min after wounding. A-B: CrMPK3-GFP fluorescence and bright field respectively. C: DAPI staining shows position of nucleus. [file 1471-2229-12-134-S5.jpeg]

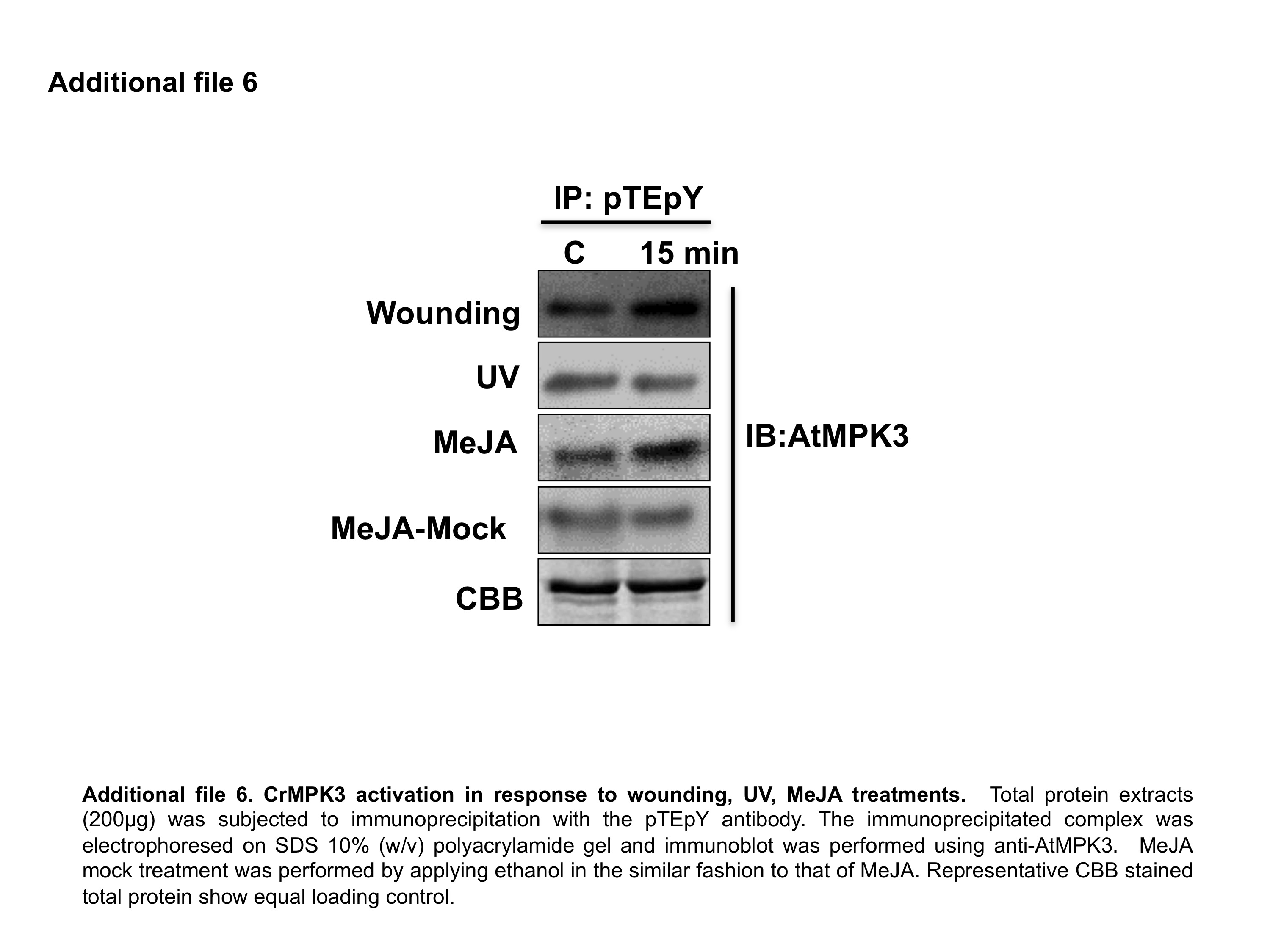

Supplement: Additional file 6 — CrMPK3 activation in response to wounding, UV, MeJA treatments. Total protein extracts (200 μg) was subjected to immunoprecipitation with the pTEpY antibody. The immunoprecipitated complex was electrophoresed on SDS 10%(w/v) polyacrylamide gel and immunoblot was performed using anti-AtMPK3. MeJA mock treatment was performed by applying solvent only (ethanol) in the similar fasion to that of MeJA. Representative CBB stained total protein show equal loading control. [file 1471-2229-12-134-S6.jpeg]

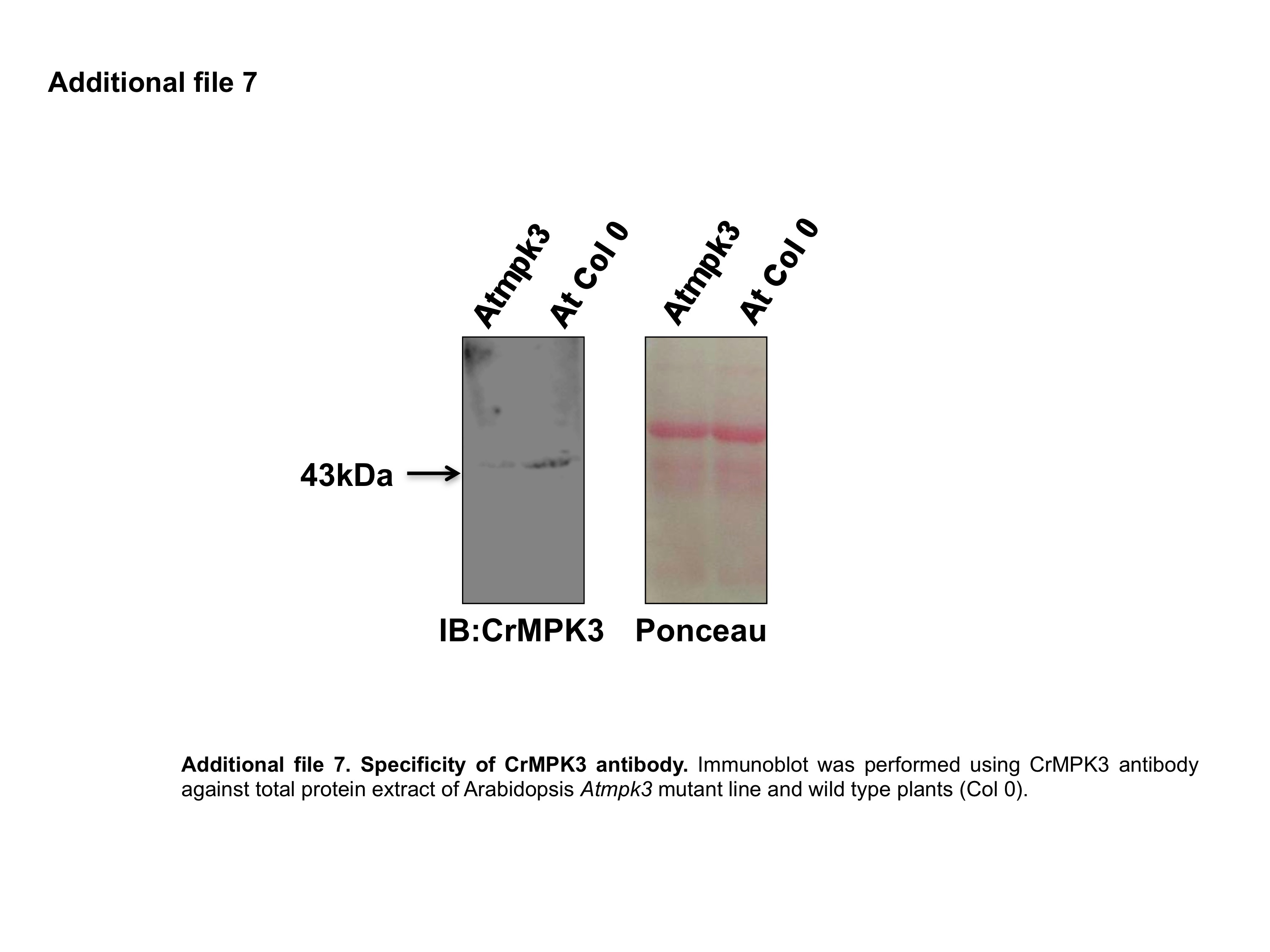

Supplement: Additional file 7 — Specificity of CrMPK3 antibody. Immunoblot was performed using CrMPK3 antibody against total protein extract of Arabidopsis Atmpk3 mutant line and wild type plants (Col 0). [file 1471-2229-12-134-S7.jpeg]
