# Supplementary material for: CrMPK3, a mitogen activated protein kinase from Catharanthus roseus and its possible role in stress induced biosynthesis of monoterpenoid indole alkaloids
Source: BMC Plant Biol. 2012 Aug 7;12:134. doi: 10.1186/1471-2229-12-134 (PMC3487899; doi:10.1186/1471-2229-12-134)
Supplement: Additional file 8 — List of genes and primer pairs for Q RT-PCR. [file 1471-2229-12-134-S8.doc]

**Additional file 8** List of genes and primer pairs for Q RT-PCR

| **Gene** | **Primer pairs** |
| --- | --- |
| *CrMPK3(Cathranthus roseus* Mitogen Activated Protein Kinase 3) | 5'ACGAAATGAGGATGCAAAAAGATAC-3  5'-TGCTAACTGCTGACGAGGGAAT-3 |
| *Str* (Strictosidine synthase) | 5’-TGCTTCACTCCCATCATTTACAGT-3’ 5’-CTGCCATCATGGATTTAGATTCAG-3’ |
| *D4h* (Desacetoxyvindoline 4-hydroxylase) | 5’-GACTTGAACTTTCATGCTGCTACAC-3’  5’-TCTCATCAAAAGCCTTCAATTCC-3’ |
| *Dat* (Acetyl CoA: deacetylvindoline 17-*O*-acetyltransferase) | 5’-AATCCCTCAGCCGCTATAACC-3’ 5’-ACGGATACGCACGTTTGGTAT-3’ |
| *Orca3* (AP2-domain DNA-binding protein) | 5’-TCGCGGCGGAGATAAGG-3’  5’-TCGTATGTACCCAACCAAATCCT-3’ |
| Zct1(Zinc finger DNA-binding protein) | 5’-CATGGGCGTGAAGAGATTCA-3’  5’-CCGACTTTAGAAAGAAGCATCAAAC-3’ |
| *Zct2* (Zinc finger DNA-binding protein) | 5’-CCGATGAAGCGTACGAGAGAA-3’  5’-TCAAGCAATTCGCCATAGTTGT-3’ |
| *Zct3* (Zinc finger DNA-binding protein) | 5’-CACCTACACCTGTTTTTCAATACGA-3’  5’-GCCCATGGCTGATCTAGATAGC-3’ |
| *CrActin* (Actin) | 5’-CTATGTTCCCAGGTATTGCAGATAGA-3’  5’-GCTGCTTGGAGCCAAAGC-3’ |
